# Neurite Orientation Dispersion and Density Imaging (NODDI) of Brain Microstructure in Adolescent Cannabis and Nicotine Use

**DOI:** 10.3390/bs14030231

**Published:** 2024-03-13

**Authors:** Alexander L Wallace, Kelly E. Courtney, Natasha E. Wade, Laura E. Hatz, Rachel Baca, Aaron Jacobson, Thomas T. Liu, Joanna Jacobus

**Affiliations:** 1 Psychiatry Department, University of California San Diego, La Jolla, CA 92093, USA; alwallace@health.ucsd.edu (A.L.W.);; 2 Center for Functional MRI and Department of Radiology, University of California San Diego, La Jolla, CA 92093, USA

**Keywords:** nicotine, cannabis, co-use, adolescence, young adulthood, white matter, neurodevelopment

## Abstract

Introduction: Despite evidence suggesting deleterious effects of cannabis and nicotine tobacco product (NTP) use on white matter integrity, there have been limited studies examining white matter integrity among users of both cannabis and nicotine. Further, updated white matter methodology provides opportunities to investigate use patterns on neurite orientation dispersion and density (NODDI) indices and subtle tissue changes related to the intra- and extra-neurite compartment. We aimed to investigate how cannabis and NTP use among adolescents and young adults interacts to impact the white matter integrity microstructure. Materials and Methods: A total of 221 participants between the ages of 16 and 22 completed the Customary Drinking and Drug Use Record (CDDR) to measure substance use, and underwent a magnetic resonance imaging (MRI) session. Participants were divided into NTP-control and NTP groupings and cannabis-control and cannabis groupings (≥26 NTP/cannabis uses in past 6 months). Tract-Based Spatial Statistics (TBSS) and two-way between-subjects ANOVA investigated the effects of NTP use group, cannabis use group, and their interaction on fractional anisotropy (FA) and NODDI indices while controlling for age and biological sex. Results: NTP use was associated with decreased FA values and increased orientation dispersion in the left anterior capsule. There were no significant effects of cannabis use or the interaction of NTP and cannabis use on white matter outcomes. Discussion: NTP use was associated with altered white matter integrity in an adolescent and young adult sample. Findings suggest that NTP-associated alterations may be linked to altered fiber tract geometry and dispersed neurite structures versus myelination, as well as differential effects of NTP and cannabis use on white matter structure. Future work is needed to investigate how altered white matter is related to downstream behavioral effects from NTP use.

## 1. Introduction

Adolescence and young adulthood mark periods of protracted neurodevelopment [1,2]. Subtle neurobiological processes associated with typical neuromaturation changes, such as synaptic pruning and white matter microstructure development, continue in humans until their mid to late twenties [3]. During this developmental window, neural substrates are sensitive to environmental influences that may alter health outcomes [4]. Substance use during this period is of great importance considering that use, including cannabis and nicotine, often starts and escalates during adolescence [5]. In 2022, 31% of high school seniors reported using cannabis and 25% reported using nicotine/tobacco products (NTP) in the past 30 days [6].

Due to the timing of these factors, studies investigating the effects of substance use on brain health using advanced neuroimaging approaches have increased over the last several decades. One key brain health outcome includes the examination of white matter tissue integrity, because white matter has the most prolonged period of development, with microstructural and architectural changes occurring well into late young adulthood [7]. White matter consists of myelinated axons of neurons that support fast communication within the brain [3]. The tracking of white matter volume and microstructural indices have shown differences not only within adult clinical populations [8,9,10] but across neurodevelopment [1,7], including in substance-using populations [11,12]. As adolescents age and their brains undergo neuronal pruning, white matter markers of tissue health (such as fractional anisotropy (FA)) increase, suggesting a better coherence and compactness of fiber tracts and, thus, better white matter integrity [13]. However, neuroimaging studies in adolescents who use substances suggest that white matter may exhibit abnormal neurodevelopmental processes [12].

Cannabis has been noted as a potential exogenous factor that may have a deleterious impact on white matter development. Cannabis acts on the endocannabinoid system, which is thought to mediate synaptic and cellular changes that influence pruning and cellular migration during adolescence [14,15]. Cross-sectional and longitudinal studies have demonstrated an association between decreased white matter integrity and adolescent and young adult cannabis use in both association and projection white matter fiber tracts [11,16,17]. Although early evidence has suggested that cannabis use leads to poorer white matter integrity (as evidenced by decreased FA values and changes in other common diffusion tensor imaging estimates) in adolescents and young adults, additional studies have found no relationship between cannabis use and white matter integrity [18,19,20], demonstrating that the nature of these relationships remains unclear.

NTP use in adolescents is also thought to impact white matter development through the chronic activation of nicotinic acetylcholine receptors [21]. It has been hypothesized that constant cholinergic stimulation may promote glial proliferation, leading to changes in white matter integrity during development [11]. Yet, there are far fewer studies as compared to cannabis and the findings are mixed, with some demonstrating increased white matter integrity among adolescents with tobacco exposure compared to their non-using peers [22]. Yet, others have found the inverse relationship [23], indicating that early nicotine use might be associated with deleterious white matter health trajectories during adolescence and young adulthood [24].

The use of both cannabis and NTPs is increasingly prevalent, with up to 37% of young adults reporting both cannabis and NTP use [25], and may result in differing outcomes compared to the use of either substance in isolation [26,27,28]. Despite these prevalence rates, few studies have examined the effects of cannabis and NTP use on neuroimaging outcomes [29]. The studies that have been completed by our laboratory show increased white matter tissue cerebral blood flow and poorer white matter integrity (i.e., decreased FA) among cannabis users without a history of nicotine use [30] and unique white matter profiles in nicotine and cannabis use groups; for example greater cannabis use was associated with greater FA in bilateral regions of the cingulum and the left fornix tracts, but only among those also reporting a history of nicotine [31]. These studies demonstrate that the interaction between cannabis and NTP use may lead to unique white matter morphometry in youth, and even introduce the possibility that NTP use may diminish or rescue the impact of cannabis use on the brain at an early age, prior to a long-term and chronic use history.

While outcomes such as FA and mean diffusivity (MD) have most commonly been used to measure white matter integrity, additional diffusion imaging techniques have been developed to help parse out the complicated structure of white matter [32]. Neurite orientation dispersion and density imaging (NODDI) is an approach to measure both intra- and extra-neurite water diffusion. NODDI provides important markers of neurite density, the concentration of tissue comprised by axons, and the orientation dispersion index (ODI), which reflects the neurite structure (i.e., the bending and fanning of axons and dendrites in white matter) [33]. These measures provide greater specificity to microstructural features compared to broad-strokes DTI measures such as FA [34], and evidence suggests that neurite density in particular may be more sensitive to changes that occur in early adolescence [35]. While some studies have utilized NODDI measures to investigate the pathology of diseases such as Alzheimer’s Disease [36] or broader psychiatric disorders [37], their use to investigate the impact of substance use on white matter health in adolescent or adult populations is largely nonexistent, except for one study exploring the impact of binge drinking in adults [38].

The current aims of the study are to investigate how cannabis and NTP use interact among adolescents and young adults and relate to lesser-studied white matter tissue health metrics. While studies remained mixed, the majority of studies have found decreased white matter integrity among adolescent users of cannabis and increased white matter integrity among adolescent NTP users, and therefore we hypothesize that cannabis use would be associated with decreased white matter integrity in measures of FA, ODI, and neurite density [17]. Inversely, we predicted that NTP use would demonstrate increased white matter integrity in all three measures based on the majority of nicotine-related findings to date [22]. Finally, we hypothesized that there would be an interaction between NTP and cannabis use in white matter health, as the strength of the relationship between cannabis and white matter integrity outcomes may be diminished (i.e., less deleterious) for those also using NTP.

## 2. Materials and Methods

### 2.1. Participants

Data for this report were culled from a recently completed study on the effects of nicotine and cannabis co-use on brain structure and function during adolescence/young adulthood. As previously reported [30,31,39], late adolescents/young adults (ages 16–22) were recruited through physical and electronic flyers at local high schools, community colleges, and four-year universities, as well as through social media sites. Potential participants completed screening via phone call to determine eligibility and establish substance use group classification at study enrollment. Recruitment and enrollment eligibility groups were determined based on past six-month cannabis and NTP use episodes and were defined as (1) frequent cannabis use only (≥1 weekly average cannabis use episode), (2) frequent NTP use only (≥1 weekly average NTP use episode), (3) frequent cannabis and NTP use (≥1 weekly average cannabis and ≥1 weekly average NTP use episode), and (4) controls (≤15 cannabis and NTP use in the past 6 months). The groups described here were for study enrollment purposes and not used for statistical analysis. Additional exclusionary criteria included current or past DSM-5 psychiatric disorder other than cannabis and/or tobacco use disorder, any lifetime illicit substance use >10 times, acute influence of alcohol or cannabis use at study visit (confirmed with breathalyzer, urine, and oral fluid toxicology), major psychiatric or medical issues, use of medications affecting the brain, MRI contraindications (e.g., implanted metal or metal braces), or history of developmental disability or prenatal substance exposure.

In order to investigate the effects of regular substance use, all participants were classified into two cannabis using groups: (1) regular cannabis uses (≥26 episodes of past 6-month cannabis use, or more than weekly, on average) and (2) cannabis controls (<26 episodes of past 6-month cannabis use). Additionally, all participants were also classified into two NTP use groups: (1) NTP use (≥26 episodes of past 6-month NTP use, more than weekly, on average) and (2) NTP controls (<26 episodes of past 6-month NTP use, less than weekly, on average). This re-grouping resulted in 221 subjects, maintained for the present analyses. Past 6-month use patterns rather than past year were used to account for more recent use. Infrequent nicotine and cannabis use were included in both control groups due to differences noted in casual substance use compared to regular use [40,41]. See Table 1 for substance use characteristics by group.

### 2.2. Procedures

Participants completed a single four-hour assessment and neuroimaging session. All participants completed informed consent protocols in adherence with the local university Institutional Review Board. Participants were asked to refrain from using cannabis and alcohol for >12 h prior to their research appointment, which was verified by urine, oral fluid, and breathalyzer testing. The Drager DrugTest^®^ 5000 tested onsite oral fluid for recent Δ9-tetrahydrocannabinol (THC) use (≥5 μg/L THC). Urine samples were sent to a toxicology lab to quantify cotinine (nicotine metabolite) and THCCOOH (THC metabolite) and to confirm that participants were negative for other substance usage. Participants were not required to abstain from NTP use to avoid any deleterious effects of nicotine withdrawal; however, self-reports of last NTP use were collected. During the research visit, participants underwent comprehensive demographic, mental health, and substance use interviews, a full neurocognitive battery, and a magnetic resonance imaging (MRI) scanning session.

### 2.3. Materials

Demographics: A psychosocial interview was conducted to obtain relative demographic variables such as age, sex assigned at birth, race/ethnicity, socioeconomic status, education, and medical history.

Substance Use: A modified version of the Customary Drinking and Drug Use Record (CDDR; [17,42]) was administered by a trained research assistant to obtain current and lifetime substance use data including cannabis and NTP use. Participants were first asked whether they had ever tried a substance in their lifetime. If they had used a substance, participants were asked how many times they had independently used cannabis products (e.g., flower; concentrates, edibles, and tinctures) and NTPs (e.g., cigarettes, cigars, vape, pipe, hookah, smokeless tobacco, and nicotine replacement). In this way, measures of past month, three-month, six-month, and past year cannabis and NTP independent use episodes were obtained.

### 2.4. Neuroimaging

Imaging studies were conducted on a 3.0 Tesla GE Discovery MR750 scanner with a Nova Medical 32-channel receive-only head coil. A high-resolution T1-weighted anatomical scan was acquired using an inversion-prepared fast spoiled gradient echo sequence with parameters TI/TE/TR = 1060/2/2500 ms, flip angle = 8°, field of view (FOV) = 256 mm, matrix = 256 × 256, 1.0 mm^3^ voxels. Diffusion data were collected with a multi-shell 96-direction single-shot spin echo diffusion sequence with b-values (500, 1000, 2000, and 3000 s/mm^2^) and 6, 15, 15, and 60 unique diffusion directions, respectively, for each b-value (TE/TR = 81.9/4100 ms, 81 axial slices, FOV = 240 mm, matrix = 140 × 140, 1.7 mm^3^ voxels). Acquisition parameters were modeled after those used in the Adolescent Brain Cognitive Development (ABCD) Study [43].

All data were visually checked for artifacts and general image quality by a trained research team member (JJ or KC). FA values were obtained using FSL’s FMRIB’s Diffusion Toolbox [44]. FSL’s TOPUP program was used to correct susceptibility-induced distortions. FSL’s eddy tool was used to correct for eddy current distortions and subject motion. FMRIB’s Linear Image Registration Tool (FLIRT) was utilized for linear registration to standard space. Finally, DTIFIT in FSL was used at the subject level to derive FA values [45]. NODDI parameter maps were obtained using the NODDI MATLAB toolbox [33]. Resulting parameter maps were used to create ODI and neurite density outputs in standard space at the subject level.

### 2.5. Analyses

Between-subject comparisons of FA, ODI, and neurite density maps were completed using FSL’s Tract-Based Spatial Statistics (TBSS; [46]). Nonlinear registration was used to determine the alignment of subject-level FA data to a standard-space image (FSL’s FMRIB58_FA). FA data were then nonlinearly transformed and merged into a single 4D image, which was used to create a mean FA tract skeleton. The FA skeleton threshold was set to 0.2 to exclude voxels containing grey matter. Each participant’s ODI and neurite density data were projected onto the tract skeleton to create concurrent mean ODI and neurite density tract skeletons. Voxel-wise statistics were then run to model a two-way between-subjects ANOVA investigating the effects of the NTP use group and cannabis use group, and their interaction (representing NTP and cannabis co-use), while controlling for age and biological sex. Threshold free cluster enhancement (TFCE) was used to correct for multiple comparisons across space [47]. All statistical decisions were made at *p* < 0.05 and all significant clusters were extracted for data visualization purposes.

## 3. Results

### 3.1. Demographics and Substance Use

Cannabis use groupings: Cannabis use groups consisted of 94 cannabis controls and 127 individuals who used cannabis regularly. Past 6-month cannabis use consumption in the cannabis use group was predominately through smoked flower (smoked flower = 100%; concentrates = 94%; edibles = 86%; tinctures = 17%). Cannabis use groups significantly differed by age (t = −2.14, *p* = 0.03), sex (χ^2^ = 8.29, *p* < 0.01), and race (χ^2^ = 10.49, *p* = 0.03). They did not significantly differ by level of education (t = −1.32, *p* = 0.19) (see Table 1). Of the 94 cannabis controls, 57.4% of participants had used cannabis in their lifetime and 48.9% had used cannabis at least once in the past 6 months. Cannabis control participants that had used cannabis in the past 6 months had, on average, only four standard cannabis use episodes in the past 6 months (M = 3.79, SD = 6.02). As expected, this was significantly lower than the average regular cannabis use group past 6-month use (t = −7.47, *p* < 0.01; M = 252.49, SD = 375.11). The regular cannabis use group also predominately reported a lifetime use of NTPs (χ^2^ = 42.58, *p* < 0.01), with 88.1% of individuals in the regular cannabis use group having used NTPs compared to 46.8% of cannabis controls. Despite this, cannabis use groupings did not significantly differ by past 6-month NTP use episodes (t = −1.42, *p* = 0.15).

Nicotine use groupings: NTP use groupings consisted of 127 NTP controls and 94 individuals who used NTPs. Past 6-month NTP use consumption was predominately through vaping (vape = 94%; cigarettes = 55%; hookah = 25%; cigars = 23%; smokeless tobacco = 17%; tobacco pipe = 6%; nicotine replacement 6%). NTP use groups significantly differed by age (t = −2.62, *p* = 0.01), sex (χ^2^ = 4.15, *p* = 0.04), and years of education (t = −1.98, *p* = 0.04). Groupings did not significantly differ by race (χ^2^ = 5.59, *p* = 0.23). In the NTP control group, 48.8% reported NTP use in their lifetime with 35.4% having used NTPs in the past 6 months. All participants in the NTP use group had tried cannabis in their lifetime (100%), with 91.5% having used cannabis in the past 6 months. Further, the NTP use group had used significantly more cannabis in the past 6 months (M = 219.53, SD = 431.61) compared to NTP controls (t = −2.73, *p* < 0.01; M = 92.80, SD = 151.23). See Table 1 for more details.

### 3.2. White Matter Integrity

**Figure 1:**
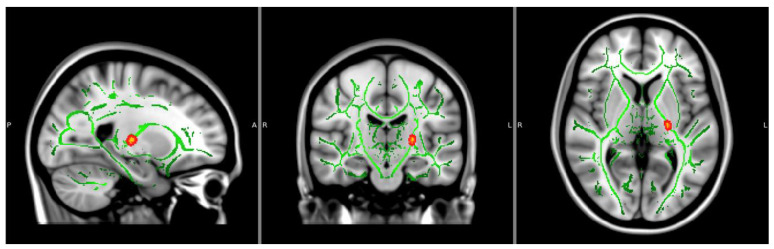
Significant FA cluster (CON > NTP, *p* < 0.05, corrected). Significant findings of the left internal capsule are displayed. Significant clusters bolded using FSL’s TBSS fill command. Axial and coronal view are in radiological view (left = right). P = Posterior; A = Anterior; R = Right; L = Left.

Fractional Anisotropy: There was a main effect of NTP use groupings on FA values within the left posterior limb of the internal capsule (*p* < 0.05, TFCE corrected), showing decreased FA values within the NTP use group compared to NTP controls, controlling for age and biological sex (see Figure 1 and Table 2). FA findings continued to be significant after extracting estimates and controlling for past year alcohol use. There were no significant effects of cannabis use groups or the interaction between NTP and cannabis use groupings.

**Figure 2:**
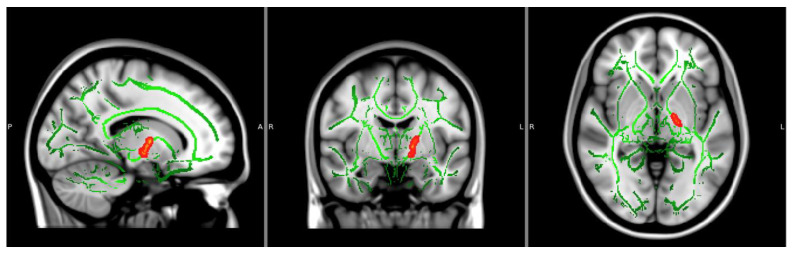
Significant OD clusters (CON < NTP, *p* < 0.05, corrected). Notes: Significant findings of the left internal capsule are displayed. Significant clusters bolded using FSL’s TBSS fill command. Axial and coronal views are in radiological view (left = right). P = Posterior; A = Anterior; R = Right; L = Left.

**Figure 3:**
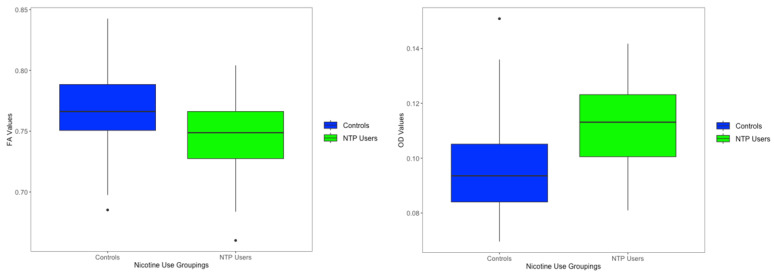
Significant FA and ODI cluster beta values (for data visualization). Notes: Boxplot represents the average beta values between both significant clusters.

Orientation Dispersion Index: There was a significant main effect of NTP use groupings on ODI values in three distinct clusters, all within the left posterior limb of the internal capsule (*p*s < 0.05, TFCE corrected), controlling for age and biological sex (see Figure 2 and Table 2). Findings showed larger ODI estimates in NTP use groups compared to the control group (see Figure 3; the figure is only for visualization purposes to help with the interpretation of results). ODI findings continued to be significant after extracting estimates and controlling for past year alcohol use. There were no significant effects of cannabis use groupings or the interaction between NTP and cannabis use groupings.

Neurite Density: There were no significant effects of cannabis use groupings, NTP use groupings, or their interaction on neurite density values.

## 4. Discussion

We aimed to investigate the relationship between NTP use group status, cannabis use status, and their interaction on white matter integrity, including white matter microstructure. Adolescent and young adult NTP use groups, compared to NTP controls, had lower FA and higher ODI values in left regions of the internal capsule. There were no significant differences in neurite density between any substance use groupings. Further, there were no significant differences in white matter integrity between cannabis use nor an interaction between cannabis and NTP use groupings.

The differences between NTP use and no use in white matter integrity within the left regions of the internal capsule have previously been noted; however, the directionality of these FA values are a departure from some findings in the literature which suggest that adolescent NTP use (mean ages 16–18) may have higher FA values compared to no NTP use [22,48,49,50]. Yet, one study has also observed lower FA values in a similar age range of young adults (mean age 21) who use NTPs [23], suggesting that our result may be uniquely related to later adolescence/young adulthood given the slightly older age of our sample (mean age 19). Our findings are also similar to research demonstrating lower FA values in adult NTP use compared to non-NTP use [11]. It is possible that the relationship between FA and NTP use varies by age and use patterns [24], perhaps contributing to mixed findings in the late adolescence and early adulthood literature. Indeed, previous studies compared individuals who used NTPs against no NTP use [48,50], while our study made comparisons between regular NTP use and NTP controls that included light to no NTP use. Nevertheless, individuals who used NTPs demonstrated lower FA values within our sample, and decreasing FA values have been linked to poor brain and behavioral outcomes across medical conditions in the developmental literature [51]. This is particularly noteworthy as FA values have been shown to increase as individuals undergo healthy neurodevelopment [13]. NTP use may continue to interrupt white matter in these areas, resulting in altered tissue integrity and increased vulnerability to addiction and pathology. Targeted and longitudinal work is needed to help decipher the exact age during neurodevelopment at which NTP use results in a high risk of white matter integrity disruption, as well as the functional outcomes related to that disruption.

A closer investigation of the white matter microstructure demonstrated higher ODI values in individuals who use NTPs compared to controls. ODI may provide a better index of the biological characteristics, such as intra- versus extra-cellular change, that are different among groups compared to the traditional DTI metrics (e.g., FA) that are less specific. Similar to our FA findings, the ODI differences were observed in the left regions of the internal capsule. While this relationship aligns with previous works in the literature demonstrating that FA values are more strongly influenced by ODI compared to neurite density and show a negative correlation from childhood to adulthood [33,35], the absence of neurite density results may also be due to the age group under study, as neurite density is thought to be sensitive to younger-age-related changes in myelin and the intracellular neurite compartment (e.g., ages 12–14; [35]). The presence of ODI differences and absence of neurite density differences in our sample also suggests that the observed differences in ODI may be due to geometrical fiber tract changes as opposed to myelination and tract packing. Together, the higher ODI, lower FA, and null neurite density findings suggest that NTP use may show increases in dispersions of fiber tracts projections (more complex bending and branching, and possibly less axonal alignment and coherence) during late adolescence and early adulthood [52]; however, whether this is related to poorer or better health outcomes is still unclear [36,37,53].

While no other studies have investigated the effects of NTP use on microstructure such as ODI, similar findings of increased ODI within the posterior limb of the internal capsule have been demonstrated in individuals diagnosed with schizophrenia [54] and major depressive disorder compared to controls [55], suggesting a link in dendritic complexity and psychopathology. Studies examining NODDI parameters among multiple sclerosis patients have found higher ODI values during acute inflammation stages [56]. Further, a study investigating adults who binge drink showed higher ODI findings compared to controls in ventral striatal and parietal grey matter regions [38]. It is possible that less aligned and more dispersed neurite structures or dendritic complexity among young adult NTP-users is related to acute inflammatory processes and/or neural vulnerability for psychopathology and addictive disorders, both of which can also have downstream neurocognitive consequences [57], particularly for motor and sensory functioning, given the involvement of the posterior limb of the internal capsule in our findings [58]. However, more work is needed to investigate the direct association between white matter microstructure and cognition before further relationships can be elucidated.

Interestingly, there were no significant relationships between cannabis use or cannabis and NTP co-use on white matter integrity observed in the present sample. Although studies of cannabis’ impact on white matter structure have provided mixed results, significant effects are typically only found with heavy cannabis use [11]. Since our sample compared individuals who regularly use cannabis (at least weekly) against individuals who engaged in light to no cannabis use (less than weekly), it is possible that heavier cannabis use in this sample has yet to significantly impact white matter structure during this particular window of neurodevelopment. Cannabis use during neurodevelopment is complex, with variables such as age of regular use [59] and duration of cannabis use [60] being important predictors of health outcomes. Further, studies from our laboratory investigating the relationship between cannabis and NTP use have suggested that individuals who use both substances may have distinct white matter phenotypes compared to cannabis use only during the 16–22-year-old age range [31].

As with all studies, there are some limitations. Our study was cross-sectional in nature, which limits the ability to determine directionality and, therefore, causation between substance use and white matter integrity. Utilizing longitudinal datasets such as the Adolescent Brain Cognitive Development (ABCD) Study [61] will be important for investigating the causality of substance use and brain health relationships. Ongoing longitudinal data collection in our laboratory utilizing NODDI-derived estimates will be examined in future investigations. Additionally, despite the fairly tight age range and restricted demographic characteristics of the full sample, it is possible that demographic and contextual differences within substance use groupings that were not measured may have influenced findings. The inclusion of a pure nicotine and tobacco product use group (i.e., without use of any other substances) may yield different findings, although the vast majority of youth nicotine product user also report cannabis use [62,63]. Further, while the comparison of individuals with regular cannabis and NTP use to a combined sample of individuals with light (less than weekly) and no substance use was carried out intentionally to explore the unique effects of heavier and more regular use compared to light use, follow-up studies investigating cannabis and NTP-only groupings against individuals who do not use substances would be important for determining if even light substance use plays a role in white matter development. Similarly, future studies modeling co-use episodes (i.e., episodes that capture simultaneous use as compared to single substance use) will help us to better understand if use at the same time has different brain health outcomes.

This is the first known study to investigate the role of cannabis and NTP co-use on ODI and neurite density estimates, in addition to FA. Our study found that NTP use, but not cannabis-only or cannabis and NTP co-use, impacted white matter integrity estimates within left regions of the posterior limb of the internal capsule in a sample of late adolescents/young adults. These findings were found not only with FA markers but with ODI as well, suggesting reduced white matter integrity at the microstructural level. These results were found within an adolescent and young adult cohort who were still undergoing neuromaturation; thus, continued changes may occur with ongoing substance use. Future longitudinal work will be important for determining the relationship between brain development and substance use as well as additional factors that may better explain the impact of substance use on white matter integrity.

## Figures and Tables

**Table 1:** Cannabis and nicotine/tobacco product (NTP) use descriptives.

	Cannabis Use (CU)			NTP Use		
M(SD)/%	Cannabis Controls (N = 94)	Cannabis Use (N = 127)	*p*-Value	NTP Controls (N = 127)	NTP Use (N = 94)	*p*-Value
Age	19.2 (1.7)	19.7(1.5)	0.03	19.3 (1.6)	19.8 (1.5)	<0.01
Sex (% Male)	57.4%	63.0%	<0.01	48.0%	62.8%	0.04
Race (% White)	47.9%	52.0%	0.03	46.5%	55.3%	0.23
Years of Education	12.9 (1.7)	13.2 (1.4)	0.18	12.9 (1.6)	13.3 (1.4)	0.05
Substance Use						
Ever Used Cannabis	57.4%	100%	<0.01	68.5%	100.0%	<0.01
Past 6-month CU	3.8 (6.0)	252.5 (375.1)	<0.01	92.8 (151.2)	219.5 (431.6)	<0.01
Days Since Last CU	103.5 (187.3) ^a^	2.7 (4.5)	<0.01	14.7 (30.6)	50.2 (152.0)	0.03
Lifetime CU Episodes	69.7 (186.3) ^a^	1145.4 (2025.2)	<0.01	382.8 (757.8)	1100.1 (2277.5)	<0.01
Age of First Regular CU	17.2 (1.3) ^b^	17.8 (1.7)	0.15	17.8 (1.6)	17.6 (1.7)	0.44
Ever Used NTP	46.8%	88.2%	<0.01	48.8%	100%	<0.01
Past 6-month NTP Use	507.7 (1477.1)	905.6 (2661.0)	0.16	2.2 (4.5)	1728.4 (3183.7)	<0.01
Lifetime NTP Episodes	69.7 (186.3)	1145.4 (2025.2)	<0.01	433.9 (1915.1)	7495.2 (14,431.5)	<0.01
Age of First Regular NTP	17.9 (1.6)	18.3 (1.7)	0.28	18.2 (1.4) ^c^	18.2 (1.7)	0.94
Ever Used Alcohol	77.7%	100%	<0.01	83.5%	100%	<0.01
Past Year Alcohol Use	34.7 (52.6)	56.2 (57.2)	<0.01	30.7 (44.1)	68.5 (63.0)	<0.01

Notes: CU = cannabis use; NTP = nicotine/tobacco product. Cannabis, NTP, and alcohol use are composites of total use derived from the assessment of standard units of each substance (cannabis = flower, concentrates, vaping, dabs, tinctures; nicotine = cigarettes, e-cigarettes, cigars, pipe, hookah, smokeless tobacco; alcohol = beer, wine, hard liquor); regular use defined as weekly use. ^a^ N = 54 to only includes cannabis control participants who had used cannabis; ^b^ N = 14 to only include cannabis control participants who had used cannabis regularly; ^c^ N = 10 to only include control NTP participants who had used NTPs regularly.

**Table 2:** Significant clusters.

**FA Values**	**Voxels**	**MAX**	**MAX X (vox)**	**MAX Y (vox)**	**MAX Z (vox)**
	22	0.95	−23	−19	2
**OD Values**	**Voxels**	**MAX**	**MAX X (vox)**	**MAX Y (vox)**	**MAX Z (vox)**
	39	0.958	−16	−11	−5
	27	0.962	−16	−5	5
	17	0.956	−21	−16	1

Notes: Voxels represent the number of voxels in each significant cluster. Max represents the maximum beta value within the cluster. MAX X/Y/Z is the location of the maximum intensity voxel. FA = fractional anisotropy. OD = orientation dispersion.

## Data Availability

The data presented in this study are available on request from the corresponding author. The data are not publicly available due to confidentiality.

## References

[1] Spear L.P. (2013). Adolescent Neurodevelopment. J. Adolesc. Health.

[2] Walker E.F. (2002). Adolescent Neurodevelopment and Psychopathology. Curr. Dir. Psychol. Sci..

[3] Lebel C., Deoni S. (2018). The Development of Brain White Matter Microstructure. NeuroImage.

[4] Squeglia L.M., Jacobus J., Tapert S.F. (2009). The Influence of Substance Use on Adolescent Brain Development. Clin. EEG Neurosci..

[5] Halladay J., Woock R., El-Khechen H., Munn C., MacKillop J., Amlung M., Ogrodnik M., Favotto L., Aryal K., Noori A. (2020). Patterns of Substance Use among Adolescents: A Systematic Review. Drug Alcohol Depend..

[6] Miech R.A., Johnston L.D., Patrick M.E., O’Malley P.M., Bachman J.G., Schulenberg J.E. (2023). Monitoring the Future National Survey Results on Drug Use, 1975–2022: Secondary School Students.

[7] Lebel C., Treit S., Beaulieu C. (2019). A Review of Diffusion MRI of Typical White Matter Development from Early Childhood to Young Adulthood. NMR Biomed..

[8] Bodini B., Ciccarelli O., Johansen-Berg H., Behrens T.E.J. (2009). CHAPTER 9—Diffusion MRI in Neurological Disorders. Diffusion MRI..

[9] Fan Y.-T., Fang Y.-W., Chen Y.-P., Leshikar E.D., Lin C.-P., Tzeng O.J.L., Huang H.-W., Huang C.-M. (2019). Aging, Cognition, and the Brain: Effects of Age-Related Variation in White Matter Integrity on Neuropsychological Function. Aging Ment. Health.

[10] Hu H.-Y., Ou Y.-N., Shen X.-N., Qu Y., Ma Y.-H., Wang Z.-T., Dong Q., Tan L., Yu J.-T. (2021). White Matter Hyperintensities and Risks of Cognitive Impairment and Dementia: A Systematic Review and Meta-Analysis of 36 Prospective Studies. Neurosci. Biobehav. Rev..

[11] Hampton W.H., Hanik I.M., Olson I.R. (2019). Substance Abuse and White Matter: Findings, Limitations, and Future of Diffusion Tensor Imaging Research. Drug Alcohol. Depend..

[12] Pando-Naude V., Toxto S., Fernandez-Lozano S., Parsons C.E., Alcauter S., Garza-Villarreal E.A. (2021). Gray and White Matter Morphology in Substance Use Disorders: A Neuroimaging Systematic Review and Meta-Analysis. Transl. Psychiatry.

[13] Madden D.J., Bennett I.J., Song A.W. (2009). Cerebral White Matter Integrity and Cognitive Aging: Contributions from Diffusion Tensor Imaging. Neuropsychol. Rev..

[14] Bara A., Ferland J.-M.N., Rompala G., Szutorisz H., Hurd Y.L. (2021). Cannabis and Synaptic Reprogramming of the Developing Brain. Nat. Rev. Neurosci..

[15] Lubman D.I., Cheetham A., Yücel M. (2015). Cannabis and Adolescent Brain Development. Pharmacol. Ther..

[16] Chye Y., Christensen E., Yücel M. (2020). Cannabis Use in Adolescence: A Review of Neuroimaging Findings. J. Dual Diagn..

[17] Jacobus J., Courtney K.E., Hodgdon E.A., Baca R. (2019). Cannabis and the Developing Brain: What Does the Evidence Say?. Birth Defects Res..

[18] Cousijn J., Wiers R.W., Ridderinkhof K.R., van den Brink W., Veltman D.J., Goudriaan A.E. (2012). Grey Matter Alterations Associated with Cannabis Use: Results of a VBM Study in Heavy Cannabis Users and Healthy Controls. NeuroImage.

[19] Orr J.M., Paschall C.J., Banich M.T. (2016). Recreational Marijuana Use Impacts White Matter Integrity and Subcortical (but Not Cortical) Morphometry. NeuroImage Clin..

[20] Thayer R.E., YorkWilliams S., Karoly H.C., Sabbineni A., Ewing S.F., Bryan A.D., Hutchison K.E. (2017). Structural Neuroimaging Correlates of Alcohol and Cannabis Use in Adolescents and Adults. Addiction.

[21] Goriounova N., Mansvelder H. (2012). Nicotine Exposure during Adolescence Alters the Rules for Prefrontal Cortical Synaptic Plasticity during Adulthood. Front. Synaptic Neurosci..

[22] Gogliettino A.R., Potenza M.N., Yip S.W. (2016). White Matter Development and Tobacco Smoking in Young Adults: A Systematic Review with Recommendations for Future Research. Drug Alcohol Depend..

[23] Kangiser M.M., Thomas A.M., Kaiver C.M., Lisdahl K.M. (2020). Nicotine Effects on White Matter Microstructure in Young Adults. Arch. Clin. Neuropsychol..

[24] Hudkins M., O’Neill J., Tobias M.C., Bartzokis G., London E.D. (2012). Cigarette Smoking and White Matter Microstructure. Psychopharmacology.

[25] Tucker J.S., Pedersen E.R., Seelam R., Dunbar M.S., Shih R.A., D’Amico E.J. (2019). Types of Cannabis and Tobacco/Nicotine Co-Use and Associated Outcomes in Young Adulthood. Psychol. Addict. Behav..

[26] Agrawal A., Lynskey M.T., Madden P.A.F., Pergadia M.L., Bucholz K.K., Heath A.C. (2009). Simultaneous Cannabis and Tobacco Use and Cannabis-Related Outcomes in Young Women. Drug Alcohol. Depend..

[27] Fairman B.J. (2015). Cannabis Problem Experiences among Users of the Tobacco-Cannabis Combination Known as Blunts. Drug Alcohol. Depend..

[28] Ream G.L., Benoit E., Johnson B.D., Dunlap E. (2008). Smoking Tobacco along with Marijuana Increases Symptoms of Cannabis Dependence. Drug Alcohol. Depend..

[29] Hernandez Mejia M., Wade N.E., Baca R., Diaz V.G., Jacobus J. (2021). The Influence of Cannabis and Nicotine Co-Use on Neuromaturation: A Systematic Review of Adolescent and Young Adult Studies. Biol. Psychiatry.

[30] Courtney K.E., Baca R., Doran N., Jacobson A., Liu T.T., Jacobus J. (2020). The Effects of Nicotine and Cannabis Co-Use during Adolescence and Young Adulthood on White Matter Cerebral Blood Flow Estimates. Psychopharmacology.

[31] Courtney K.E., Sorg S., Baca R., Doran N., Jacobson A., Liu T.T., Jacobus J. (2022). The Effects of Nicotine and Cannabis Co-Use During Late Adolescence on White Matter Fiber Tract Microstructure. J. Stud. Alcohol. Drugs.

[32] Assaf Y., Cohen Y., Johansen-Berg H., Behrens T.E.J. (2014). Chapter 9—Inferring Microstructural Information of White Matter from Diffusion MRI. Diffusion MRI.

[33] Zhang H., Schneider T., Wheeler-Kingshott C.A., Alexander D.C. (2012). NODDI: Practical in Vivo Neurite Orientation Dispersion and Density Imaging of the Human Brain. NeuroImage.

[34] Timmers I., Roebroeck A., Bastiani M., Jansma B., Rubio-Gozalbo E., Zhang H. (2016). Assessing Microstructural Substrates of White Matter Abnormalities: A Comparative Study Using DTI and NODDI. PLoS ONE.

[35] Mah A., Geeraert B., Lebel C. (2017). Detailing Neuroanatomical Development in Late Childhood and Early Adolescence Using NODDI. PLoS ONE.

[36] Colgan N., Siow B., O’Callaghan J.M., Harrison I.F., Wells J.A., Holmes H.E., Ismail O., Richardson S., Alexander D.C., Collins E.C. (2016). Application of Neurite Orientation Dispersion and Density Imaging (NODDI) to a Tau Pathology Model of Alzheimer’s Disease. NeuroImage.

[37] Kraguljac N.V., Guerreri M., Strickland M.J., Zhang H. (2023). Neurite Orientation Dispersion and Density Imaging in Psychiatric Disorders: A Systematic Literature Review and a Technical Note. Biol. Psychiatry Glob. Open Sci..

[38] Morris L.S., Dowell N.G., Cercignani M., Harrison N.A., Voon V. (2018). Binge Drinking Differentially Affects Cortical and Subcortical Microstructure. Addict. Biol..

[39] Wade N.E., Baca R., Courtney K.E., McCabe C.J., Infante M.A., Huestis M.A., Jacobus J. (2021). Preliminary Evidence for Cannabis and Nicotine Urinary Metabolites as Predictors of Verbal Memory Performance and Learning Among Young Adults. J. Int. Neuropsychol. Soc..

[40] Callaghan R.C., Sanches M., Kish S.J. (2020). Quantity and Frequency of Cannabis Use in Relation to Cannabis-Use Disorder and Cannabis-Related Problems. Drug Alcohol. Depend..

[41] Wang Y., Sung H.-Y., Yao T., Lightwood J., Max W. (2018). Infrequent and Frequent Nondaily Smokers and Daily Smokers: Their Characteristics and Other Tobacco Use Patterns. Nicotine Tob. Res..

[42] Brown S.A., Myers M.G., Lippke L., Tapert S.F., Stewart D.G., Vik P.W. (1998). Psychometric Evaluation of the Customary Drinking and Drug Use Record (CDDR): A Measure of Adolescent Alcohol and Drug Involvement. J. Stud. Alcohol.

[43] Hagler D.J., Hatton S., Cornejo M.D., Makowski C., Fair D.A., Dick A.S., Sutherland M.T., Casey B.J., Barch D.M., Harms M.P. (2019). Image Processing and Analysis Methods for the Adolescent Brain Cognitive Development Study. NeuroImage.

[44] Behrens T.E.J., Berg H.J., Jbabdi S., Rushworth M.F.S., Woolrich M.W. (2007). Probabilistic Diffusion Tractography with Multiple Fibre Orientations: What Can We Gain?. NeuroImage.

[45] Behrens T.E.J., Woolrich M., Jenkinson M., Johansen-Berg H., Nunes R., Clare S., Matthews P., Brady J., Smith S. (2003). Characterization and Propagation of Uncertainty in Diffusion-Weighted MR Imaging. Magn. Reson. Med..

[46] Smith S.M., Jenkinson M., Johansen-Berg H., Rueckert D., Nichols T.E., Mackay C.E., Watkins K.E., Ciccarelli O., Cader M.Z., Matthews P.M. (2006). Tract-Based Spatial Statistics: Voxelwise Analysis of Multi-Subject Diffusion Data. NeuroImage.

[47] Smith S.M., Nichols T. (2009). Threshold-Free Cluster Enhancement: Addressing Problems of Smoothing, Threshold Dependence and Localisation in Cluster Inference. NeuroImage.

[48] Jacobsen L.K., Picciotto M.R., Heath C.J., Frost S.J., Tsou K.A., Dwan R.A., Jackowski M.P., Constable R.T., Mencl W.E. (2007). Prenatal and Adolescent Exposure to Tobacco Smoke Modulates the Development of White Matter Microstructure. J. Neurosci..

[49] van Ewijk H., Groenman A.P., Zwiers M.P., Heslenfeld D.J., Faraone S.V., Hartman C.A., Luman M., Greven C.U., Hoekstra P.J., Franke B. (2015). Smoking and the Developing Brain: Altered White Matter Microstructure in Attention-Deficit/Hyperactivity Disorder and Healthy Controls. Hum. Brain Mapp..

[50] Yu D., Yuan K., Zhang B., Liu J., Dong M., Jin C., Luo L., Zhai J., Zhao L., Zhao Y. (2016). White Matter Integrity in Young Smokers: A Tract-Based Spatial Statistics Study. Addict. Biol..

[51] Filley C.M., Fields R.D. (2016). White Matter and Cognition: Making the Connection. J. Neurophysiol..

[52] Jeurissen B., Leemans A., Tournier J.-D., Jones D., Sijbers J. (2010). Estimating the Number of Fiber Orientations in Diffusion MRI Voxels: A Constrained Spherical Deconvolution Study. Proc. Intl. Soc. Mag. Reson. Med..

[53] Andica C., Kamagata K., Kirino E., Uchida W., Irie R., Murata S., Aoki S. (2021). Neurite Orientation Dispersion and Density Imaging Reveals White Matter Microstructural Alterations in Adults with Autism. Mol. Autism.

[54] Kraguljac N.V., Anthony T., Monroe W.S., Skidmore F.M., Morgan C.J., White D.M., Patel N., Lahti A.C. (2019). A Longitudinal Neurite and Free Water Imaging Study in Patients with a Schizophrenia Spectrum Disorder. Neuropsychopharmacology.

[55] Ota M., Noda T., Sato N., Hidese S., Teraishi T., Setoyama S., Sone D., Matsuda H., Kunugi H. (2018). The Use of Diffusional Kurtosis Imaging and Neurite Orientation Dispersion and Density Imaging of the Brain in Major Depressive Disorder. J. Psychiatr. Res..

[56] Sacco S., Caverzasi E., Papinutto N., Cordano C., Bischof A., Gundel T., Cheng S., Asteggiano C., Kirkish G., Mallott J. (2020). Neurite Orientation Dispersion and Density Imaging for Assessing Acute Inflammation and Lesion Evolution in MS. Am. J. Neuroradiol..

[57] Swan G.E., Lessov-Schlaggar C.N. (2007). The Effects of Tobacco Smoke and Nicotine on Cognition and the Brain. Neuropsychol. Rev..

[58] Emos M.C., Khan Suheb M.Z., Agarwal S. (2022). Neuroanatomy, Internal Capsule. StatPearls.

[59] Lisdahl K. (2013). Dare to Delay? The Impacts of Adolescent Alcohol and Marijuana Use Onset on Cognition, Brain Structure, and Function. Front. Psychiatry.

[60] Filbey F.M., Aslan S., Calhoun V.D., Spence J.S., Damaraju E., Caprihan A., Segall J. (2014). Long-Term Effects of Marijuana Use on the Brain. Proc. Natl. Acad. Sci. USA.

[61] Volkow N.D., Koob G.F., Croyle R.T., Bianchi D.W., Gordon J.A., Koroshetz W.J., Pérez-Stable E.J., Riley W.T., Bloch M.H., Conway K. (2018). The Conception of the ABCD Study: From Substance Use to a Broad NIH Collaboration. Dev. Cogn. Neurosci..

[62] Ramo D.E., Prochaska J.J. (2012). Prevalence and Co-Use of Marijuana among Young Adult Cigarette Smokers: An Anonymous Online National Survey. Addict. Sci. Clin. Pract..

[63] Keyes K.M., Kreski N.T., Ankrum H., Cerdá M., Chen Q., Hasin D.S., Martins S.S., Olfson M., Miech R. (2022). Frequency of Adolescent Cannabis Smoking and Vaping in the United States: Trends, Disparities and Concurrent Substance Use, 2017–2019. Addiction.

